# ARF6 mediates nephrin tyrosine phosphorylation-induced podocyte cellular dynamics

**DOI:** 10.1371/journal.pone.0184575

**Published:** 2017-09-07

**Authors:** Jamie S. Lin, Jin Seok Jeon, Qingfeng Fan, Hetty N. Wong, Matthew B. Palmer, Lawrence B. Holzman

**Affiliations:** 1 Division of Internal Medicine, Department of Emergency Medicine, Section of Nephrology, The University of Texas MD Anderson Cancer Center, Houston, Texas, United States of America; 2 Department of Medicine, Division of Nephrology, Soonchunhyang University Hospital, Seoul, Korea; 3 Department of Medicine, Renal-Electrolyte and Hypertension Division, Perelman School of Medicine at the University of Pennsylvania, Philadelphia, Pennsylvania, United States of America; 4 Department of Pathology and Laboratory Medicine, Hospital of the University of Pennsylvania, Philadelphia, Pennsylvania, United States of America; University of Houston, UNITED STATES

## Abstract

ADP-ribosylation factor 6 (ARF6) is a small GTPase necessary for regulating cellular structure, motility, and vesicle trafficking. In several cellular systems, ARF6 was shown to regulate actin dynamics in coordination with Rac1, a Rho small GTPase. We examined the function of ARF6 in the kidney podocyte because Rac1 was implicated in kidney diseases involving this cell. We found that ARF6 expression was enriched in human podocytes and that it modulated podocyte cytoskeletal dynamics through a functional interaction with nephrin, an intercellular junction protein necessary for podocyte injury-induced signaling requiring activation by tyrosine phosphorylation of its cytoplasmic domain. ARF6 was necessary for nephrin activation-induced ruffling and focal adhesion turnover, possibly by altering Rac1 activity. In podocyte-specific *Arf6* (ARF6_PodKO) knockout mice, ARF6 deficiency did not result in a spontaneous kidney developmental phenotype or proteinuria after aging. However, ARF6_PodKO mice exhibited distinct phenotypes in two *in vivo* glomerular injury models. In the protamine sulfate perfusion model, which induced acute podocyte effacement, ARF6_PodKO mice were protected from podocyte effacement. In the nephrotoxic serum nephritis model, which induced immune-complex mediated injury, ARF6_PodKO mice exhibited aggravated proteinuria. Together, these observations suggest that while ARF6 is necessary for nephrin tyrosine phosphorylation-induced cytoskeletal dynamics in cultured podocytes, ARF6 has pleotropic podocyte roles *in vivo*, where glomerular injury-specific mechanisms might activate distinct signaling pathways that dictate whether ARF6 activity is beneficial or deleterious for maintaining the integrity of the glomerular filtration barrier.

## Introduction

Podocyte injury is seen in nearly all human glomerular diseases and associated with functional kidney decline [1–5]. Podocytes are terminally differentiated glomerular epithelial cells that form the tripartite glomerular filtration barrier with the basement membrane and fenestrated endothelial cells and encircles glomerular capillaries with foot processes that interdigitate with those of neighboring podocytes, creating intercellular junctions comprised of protein complexes necessary to maintain the permeability characteristics of the glomerular filter [6–8]. Stress or injury to the podocyte triggers intracellular signaling events that ultimately result in actin cytoskeleton rearrangement and foot process spreading and retraction known as effacement [1, 2, 9]. This morphologic stress response of the podocyte leads to functional kidney decline and is associated with glomerular diseases such as focal segmental glomerulosclerosis, minimal change disease, and immune-complex mediated glomerulonephritis [3, 4, 10–13].

One of the key perturbations resulting in podocyte effacement is altered function of nephrin, an essential podocyte protein and a critical component of the intercellular slit diaphragm protein complex [7, 14, 15]. Human mutations in the *NPHS1* gene, which encodes nephrin, are associated with congenital nephrotic syndrome of the Finnish type [16, 17]; alterations in expression, location, or phosphorylation status of nephrin have been observed in human glomerular diseases and animal podocyte-injury models [14, 16, 18, 19]. Nephrin’s immunoglobulin-like extracellular structure consists of eight IgG motifs and a type III fibronectin domain while its intracellular domain contains ten highly-conserved tyrosine residues [17]. It is proposed that signals are propagated through nephrin’s cytoplasmic tail, where phosphorylation of these conserved tyrosine residues by Fyn kinase recruits actin adaptor proteins [20, 21] such as the p85 subunit of phosphoinositide 3-kinase (PI3K) [22–24], the Cas-Crk complex [25, 26], and Nck1/2 [19, 27]. Nephrin tyrosine phosphorylation-mediated increase in PI3K enzymatic activity subsequently increases activity of Rac1, a Rho family small GTPase that plays a pivotal role in membrane ruffling, cell motility, and actin organization [22, 24, 28]. Aberrations in Rac1 activity are associated with human minimal change disease and idiopathic focal segmental glomerulosclerosis [12, 28–31].

Nephrin tyrosine phosphorylation (i.e. nephrin activation) triggers cytoskeletal dynamics associated with increased lamellipodia activity [25, 26, 32] and focal adhesion (FA) turnover [25, 26]. These events are in part Rac1-induced [22, 23] and necessary for podocyte cytoskeletal remodeling and effacement. Because our understanding of injury-triggered mechanisms that result in podocyte effacement remains incomplete, our attention was drawn to a family of small GTPases not previously studied in human podocytes. The ADP-ribosylation factor (ARF) family which consists of ARF1-6 belongs to the Ras superfamily and is known to be involved in vesicle trafficking and cellular morphology [33–35]. Similar to all small GTPases, the ARFs function like a molecular switch where guanine exchange factors (GEFs) catalyze the exchange of GDP to GTP, thereby increasing ARF activity, whereas GTPase activating proteins (GAPs) catalyze the exchange of GTP to GDP rendering ARFs inactive [35]. Importantly, ARF6 is a key regulator of actin organization that functions in coordination with Rac1 [36–39]. However, the mechanistic link between ARF6 and the proteinuric diseases associated with Rac1 in podocyte biology has not been fully explored.

To address this gap, we sought to examine the role of ARF6 in podocytes using our well-established nephrin-activated human podocyte cell culture model and our engineered podocyte-specific ARF6 knockout (ARF6_PodKO) mouse. The aims of this study were to investigate the role of ARF6 in human podocytes in context of nephrin tyrosine phosphorylation-induced podocyte cytoskeletal dynamics and to interrogate the effects of ARF6 deficiency in acute glomerular injury *in vivo*.

## Results

### ARF6 is expressed in human and rodent podocytes *in vivo*

**Fig 1:**
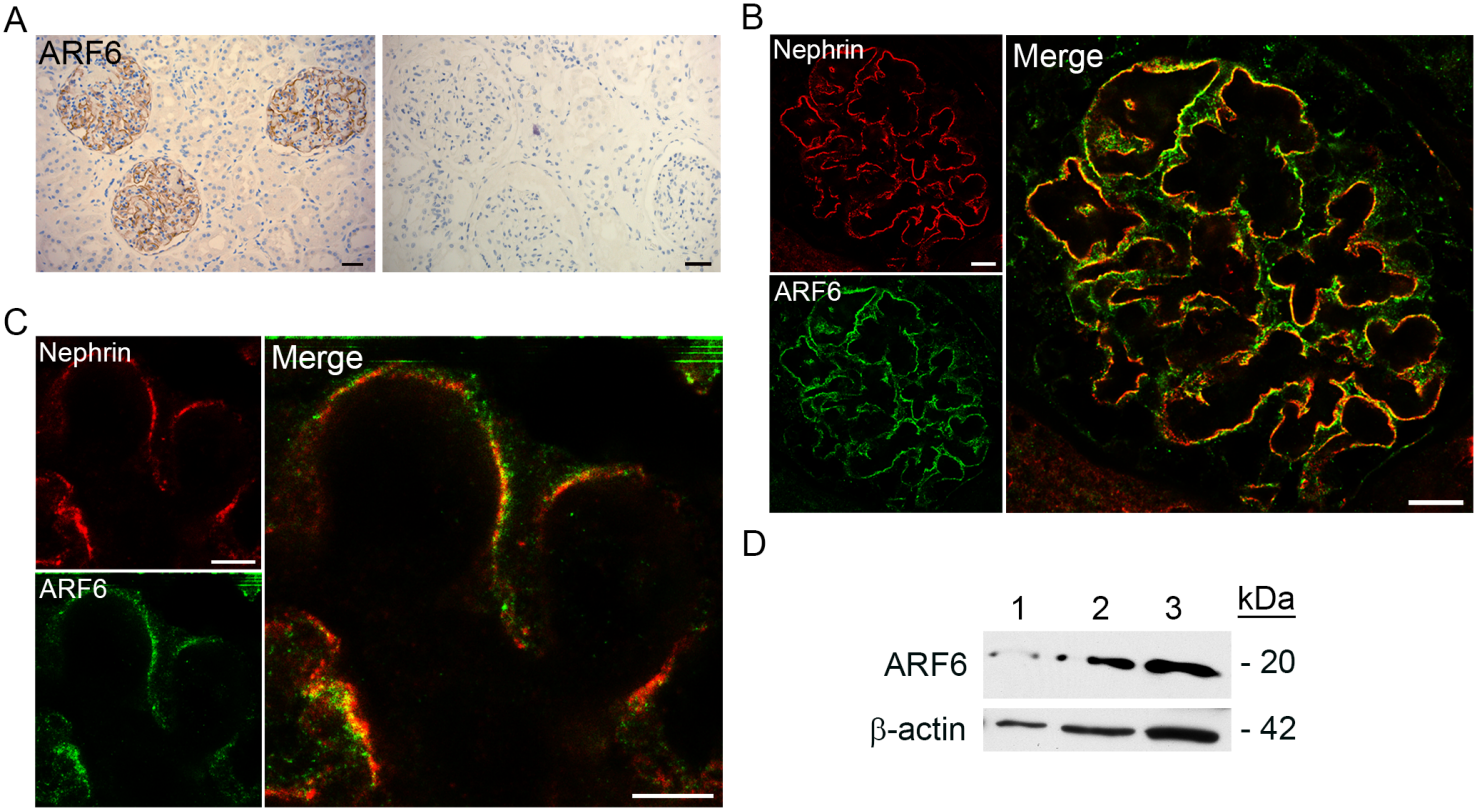
ARF6 is enriched in human glomerular podocytes. A. Sections of normal human kidney obtained by nephrectomy were stained by immunohistochemistry for ARF6 (brown). Negative control (right). Scale bar 30 μm. B. Indirect immunofluorescence confocal microscopy was used to image normal human kidney tissue stained for ARF6 (green) and nephrin (red). Magnification x63. Scale bar 11μm. C. Stimulated emission depletion micrograph of normal human kidney tissue stained for ARF6 (green) and nephrin (red). Scale bar 5 μm. D. Immunoblot analysis of ARF6 from lysate prepared from 1) normal wild-type (WT) mouse kidney, 2) isolated rat glomeruli, and 3) human podocyte cell line. β-actin was used as loading control.

ARF6 expression was present in glomeruli of normal human kidney tissue sections. Immunohistochemistry of these sections demonstrated robust ARF6 expression along the glomerular basement membrane (Fig 1A). By immunofluorescence imaging, ARF6 co-localized with nephrin at the intercellular junction of podocyte foot processes, but was also distributed to other regions of the cell body (Fig 1B and 1C). Immunoblotting confirmed the presence of ARF6 protein in whole kidney tissue lysate from wild-type (WT) mice, isolated rat glomerular lysate, and cultured human podocyte lysate. ARF6 protein was of the expected relative molecular mass of ~20 kDa (Fig 1D).

### Nephrin tyrosine phosphorylation increases ARF6 activity and nephrin-ARF6 association

**Fig 2:**
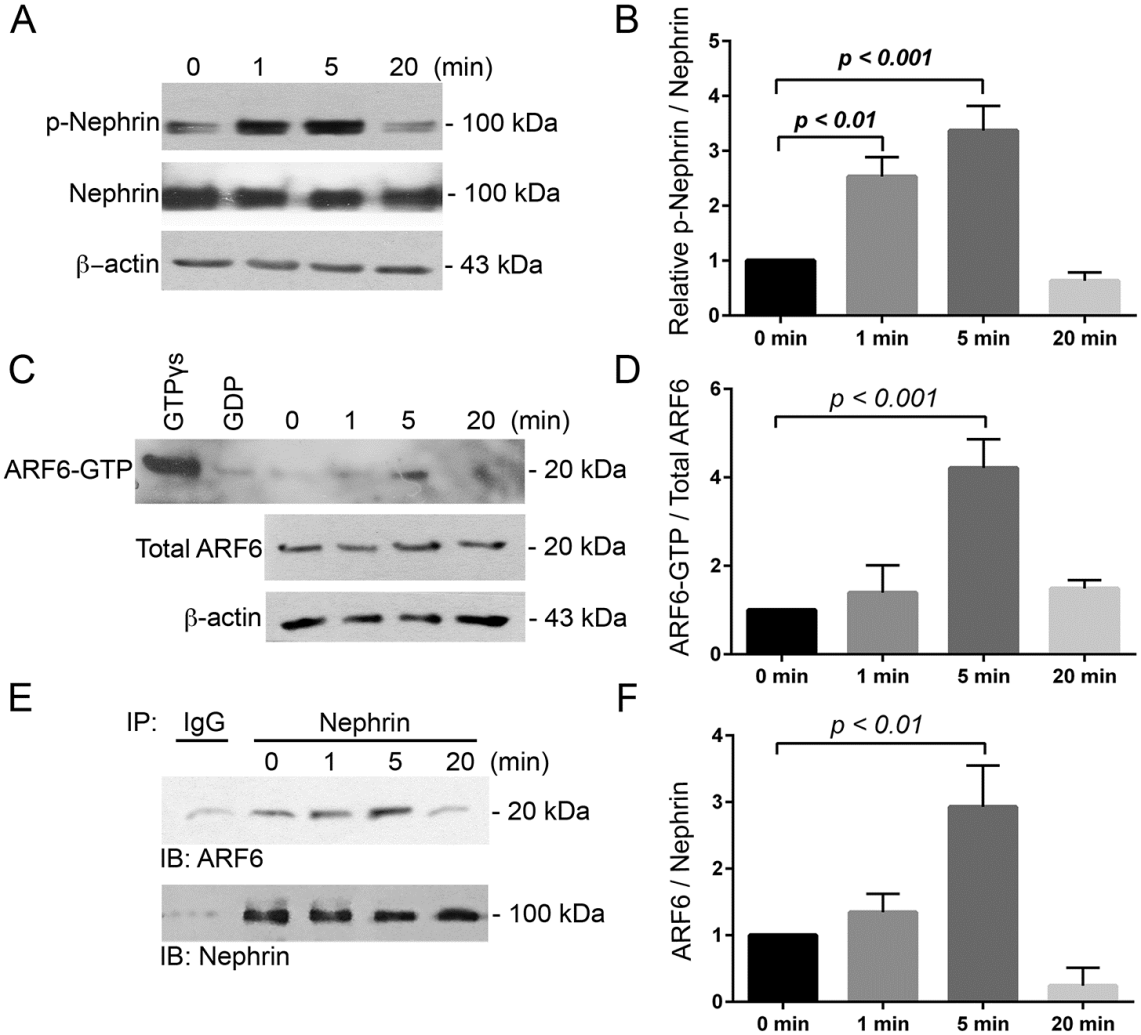
Nephrin tyrosine phosphorylation increased ARF6 activity and nephrin-ARF6 association. A. Immunoblot (IB) of total nephrin and tyrosine-phosphorylated nephrin (p-Nephrin) using the CD16/7-nephrin activation model in CD16/7-nephrin expressing human WT podocytes. B. Densitometry analysis of p-nephrin to total nephrin normalized to 0 minutes. Data are shown as mean ± SEM. C. Following nephrin induction in WT podocytes ARF6 activity was assessed by an ARF6 activation assay. GTPγs- and GDP-treated samples were used as positive and negative controls, respectively. D. Densitometry analysis of ARF6-GTP to total ARF6 normalized to 0 minutes. E. CD16/7-nephrin-ARF6 association was assessed by co-immunoprecipitation (IP) where nephrin was immunoprecipitated from podocyte lysates, and nephrin and ARF6 were detected by IB. IP using rabbit IgG was employed as control. F. Densitometry analysis of ARF6 normalized to nephrin. Assays were repeated in triplicate.

Nephrin tyrosine phosphorylation determines intracellular podocyte signaling and cytoskeletal remodeling [15, 19, 21, 24–26, 32, 40]. The potential nephrin-ARF6 relationship was examined in an *in vitro* model using chimeric nephrin protein, CD16/7-nephrin (CD16 extracellular/CD7 transmembrane domain fused to the wild-type nephrin cytoplasmic domain). The CD16/7-nephrin activation model allows for precisely-timed induction of nephrin tyrosine phosphorylation in live cells by addition of specific antibodies that cluster the extracellular CD16 domain and results in intracellular tyrosine phosphorylation of nephrin’s cytoplasmic tail. This reproducible model enables investigation of nephrin phosphorylation-mediated signaling events to be studied in detailed [25, 26, 41]. We induced nephrin tyrosine phosphorylation in human podocytes expressing CD16/7-nephrin and detected increased phosphorylation at 1 and 5 minutes (Fig 2A and 2B), reproducing previously published work [24–26]. ARF6 activity following nephrin activation was measured using an ARF6 activation assay (described in Methods). Prior to nephrin induction (0 minutes), activated ARF6 (ARF6-GTP) was detected only at a minimal level (Fig 2C and 2D). The abundance of activated ARF6 increased 5 minutes after nephrin induction (Fig 2C and 2D). Concurrent with ARF6 activation at 5 minutes, we observed an increase in nephrin-ARF6 association by co-immunoprecipitation of nephrin and ARF6 (Fig 2E and 2F).

### Podocytes depleted of ARF6 have attenuated nephrin tyrosine phosphorylation-induced ruffling

**Fig 3:**
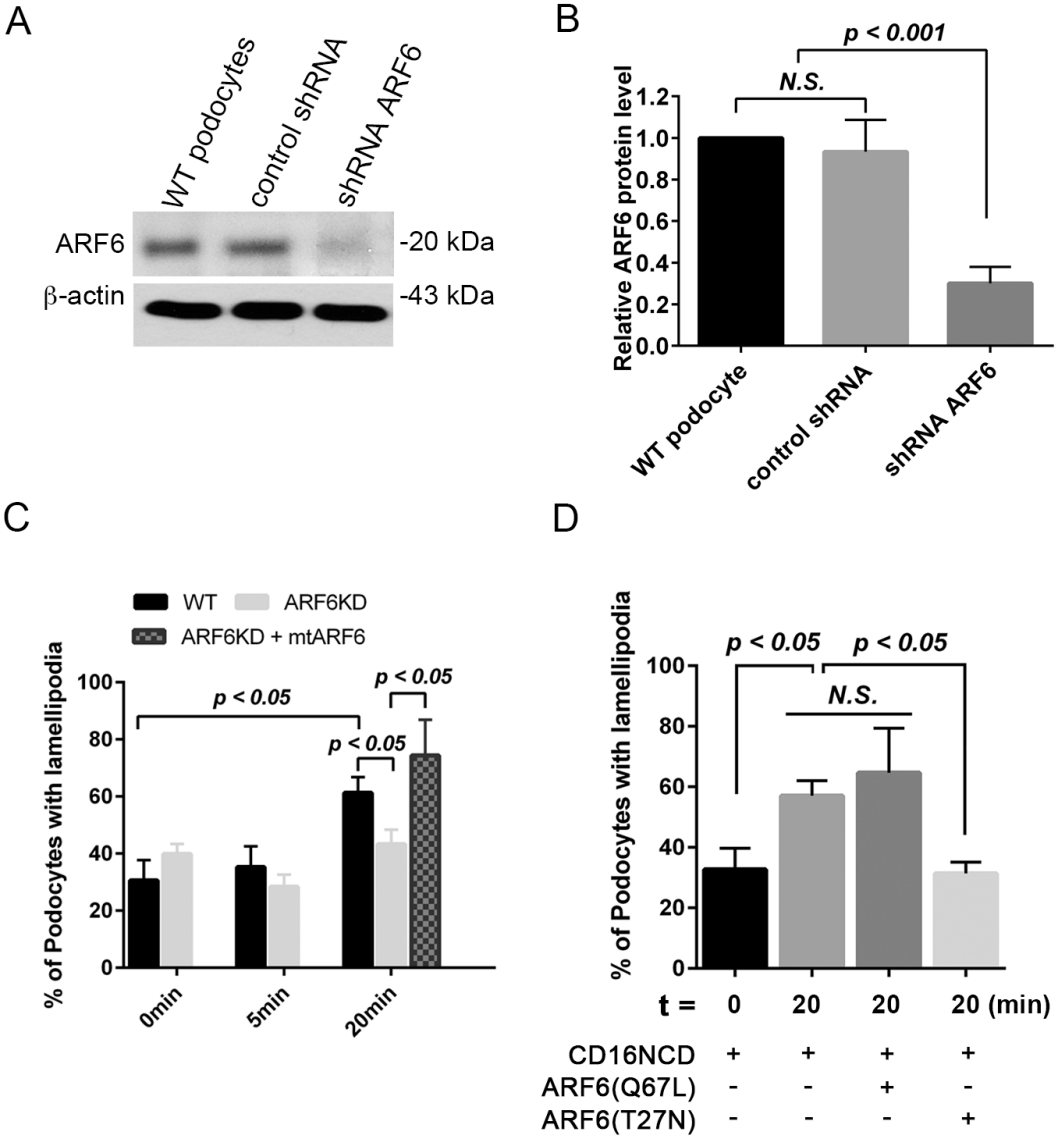
ARF6 is necessary for nephrin ligation-induced ruffling activity. A. IB of ARF6 protein expression in human WT podocytes, WT podocytes transduced with shRNA control, or *ARF6* shRNA. B. Densitometry analysis of ARF6 protein abundance normalized to β-actin. C. Percent of podocytes with positive anti-lamellipodin staining following activation of CD16/7-nephrin expressing WT and ARF6 depleted cells. Expression of mutant ARF6 (mtARF6, in which four same-sense mutations were placed within the wild type human ARF6 shRNA target region) was used to rescue ruffling activity in cultured activated CD16/7-nephrin expressing stable ARF6 KD podocytes. D. Percent of podocytes with positive anti-lamellipodin staining 20 minutes following activation of CD16/7 expressing WT, constitutively-active ARF6, ARF6(Q67L), or dominant-negative, ARF6(T27N) podocytes. Approximately 100 cells were evaluated per condition.

Nephrin activation-induced ruffling is proposed to be a cell-culture surrogate [25, 26] for podocyte effacement *in vivo*. Our prior work demonstrated that nephrin tyrosine phosphorylation is sufficient and necessary to increase lamellipodia activity [25, 26, 32, 40]; a cellular phenotype that results in increased ruffling and, by extension, is hypothesized to potentiate foot-process effacement. To investigate how ARF6 functions in nephrin phosphorylation pathways, we first sought to determine if ARF6 loss-of-function alters nephrin activation-induced podocyte ruffling *in vitro*. After treating human podocytes with either *ARF6* shRNA to induce ARF6 knockdown (ARF6KD) or non-targeting shRNA (Fig 3A and 3B), lamellipodia (ruffling) activity was assessed by indirect immunofluorescence microscopy as described in Methods (Fig 3C). Using the CD16/7-nephrin activation model, we assessed if ARF6 was necessary for nephrin tyrosine phosphorylation-mediated increase in podocyte ruffling. At 0 minutes 30.7 ± 4% of CD16/7-nephrin wild type podocytes had ruffling activity (Fig 3C, Panel A in S1 Fig). Activation of these podocytes resulted in increased ruffling activity (61.3 ± 2.7%) by 20 minutes (Fig 3C, Panel A in S1 Fig). Nephrin tyrosine phosphorylation-induced increased ruffling activity was not significantly increased at 20 min in ARF6KD podocytes expressing activated CD16/7-nephrin, but following rescue of ARF6, ruffling activity (74.3 ± 12.5%) was again induced (Fig 3C, S2 Fig and Panel B in S1 Fig).

To confirm the role of ARF6 in nephrin phosphorylation-mediated increased ruffling activity, we expressed dominant-negative ARF6, ARF6(T27N), or constitutively-active ARF6, ARF6(Q67L), variant in CD16/7-nephrin WT podocytes and investigated ruffling activity following nephrin activation. Expression of dominant-negative ARF6, ARF6(T27N), blocked nephrin tyrosine phosphorylation-mediated increased ruffling, while expression of constitutively-active ARF6, ARF6(Q67L), augmented nephrin-induced ruffles (Fig 3D, Panel C in S1 Fig). Taken together, these results suggest that ARF6 is necessary for nephrin tyrosine phosphorylation signaling-mediated increased ruffling activity.

### ARF6KD inhibits nephrin tyrosine phosphorylation mediated focal adhesion turnover

**Fig 4:**
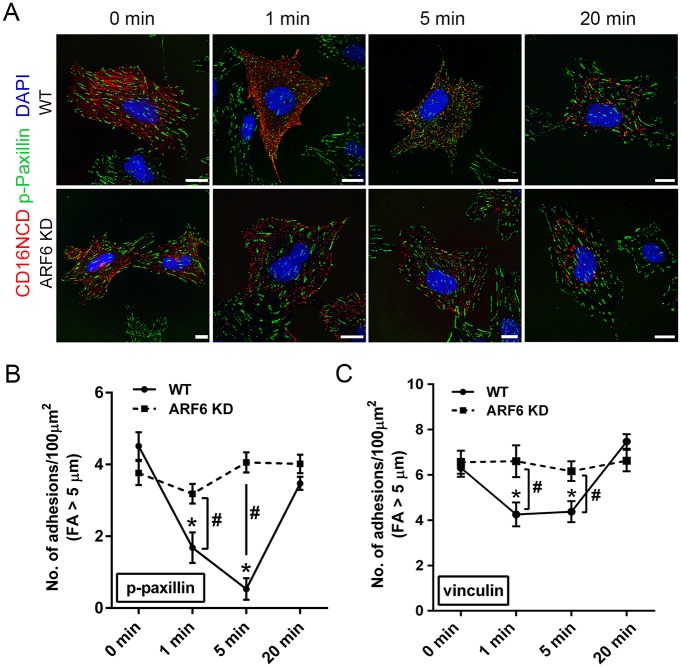
ARF6KD inhibits nephrin activation-induced dynamic focal adhesion turnover. A. CD16/7-nephrin (red) expressing WT and stable ARF6-depleted (ARF6KD) human podocytes were used to assess focal adhesion (FA) turnover following CD16/7-nephrin induction. Indirect immunofluorescence microscopy of WT and ARF6KD cells showing CD16/7-nephrin (red) and FA, phosphorylated-paxillin (p-paxillin, green), at indicated time-points following nephrin activation. Magnification 63x. Scale bar 20 μm. B. Quantification of the number of mature FA (≥5 μm Feret’s Diameter) per 100 μm^2^ cell surface area identified by p-paxillin staining at indicated times following nephrin activation. C. Quantification of the number of mature FA (≥5 μm Feret’s Diameter) per 100 μm^2^ cell surface area identified by vinculin staining at indicated times following nephrin activation.

During cell migration, focal adhesion turnover occurs where nascent focal adhesions (FA) appear in lamellipodia and elongate to mature FA slowing down actin retrograde flow. FA lengthening increases traction between the cell and extracellular matrix halting migration, thus recreating a stationary cell phenotype [42–44]. Given our observation above that ARF6 regulates ruffling, we hypothesized that ARF6 is necessary for nephrin phosphorylation-induced FA turnover. To test this hypothesis, we examined the change in density of mature FA, phosphorylated-paxillin (p-paxillin) and vinculin, over time in the CD16/7-nephrin activation model by quantifying the density of mature FA (≥ 5 μm in length) in cultured activated CD16/7-nephrin expressing WT and ARF6KD podocytes [25, 32]. Induction of CD16/7-nephrin resulted in FA turnover as seen by a time-dependent reduction within 5 minutes and recovery of p-paxillin (Fig 4A and 4B) and vinculin (Fig 4C) with positive mature FA 20 minutes post-induction. ARF6 knockdown inhibited FA turnover with no reduction in mature FA after CD16/7-nephrin induction (Fig 4A, 4B and 4C).

### Nephrin tyrosine phosphorylation-induced Rac1 activity is attenuated by ARF6 depletion

**Fig 5:**
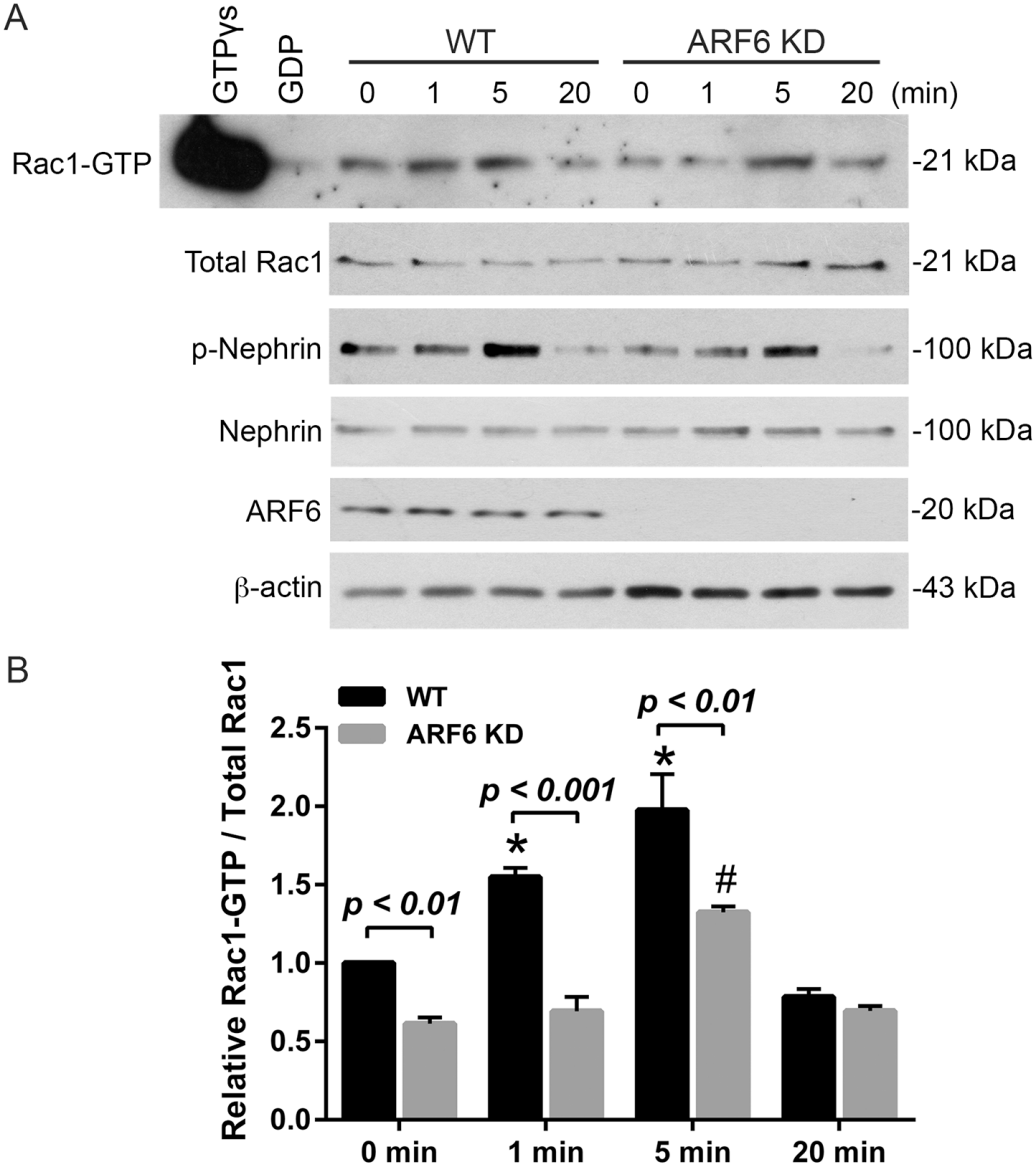
ARF6KD attenuated nephrin tyrosine phosphorylation-induced Rac1 activity. A. Following nephrin activation in CD16/7-nephrin expressing human WT or ARF6KD podocytes, Rac1 activity was determined as described in Methods. GTPγs and GDP treated samples were used as positive and negative control, respectively. B. Densitometry analysis of Rac1-GTP to total Rac1 normalized to time 0 minutes.

Nephrin signaling-induced podocyte cytoskeletal remodeling is partially mediated by increased Rac1 activity and results in podocyte effacement [22, 28, 29, 31, 45–47]. In other cell models ARF6 modulates Rac1 activity effecting cellular architecture [36, 37, 39, 48, 49]. To determine whether ARF6 is an intermediary in nephrin tyrosine phosphorylation-triggered changes to Rac1 activity, Rac1-GTP levels were investigated using a Rac1 activation assay following nephrin activation in CD16/7-nephrin expressing WT and ARF6KD podocytes. As previously observed, induction of CD16/7-nephrin WT podocytes resulted in increased Rac1 activity 1 and 5 minutes following nephrin induction [22, 28, 45], while CD16/7-nephrin podocytes depleted of ARF6 had overall decreased Rac1 activity (Fig 5A and 5B).

### Podocyte-specific ARF6 null mice have no discernable morphological or functional renal abnormalities

**Fig 6:**
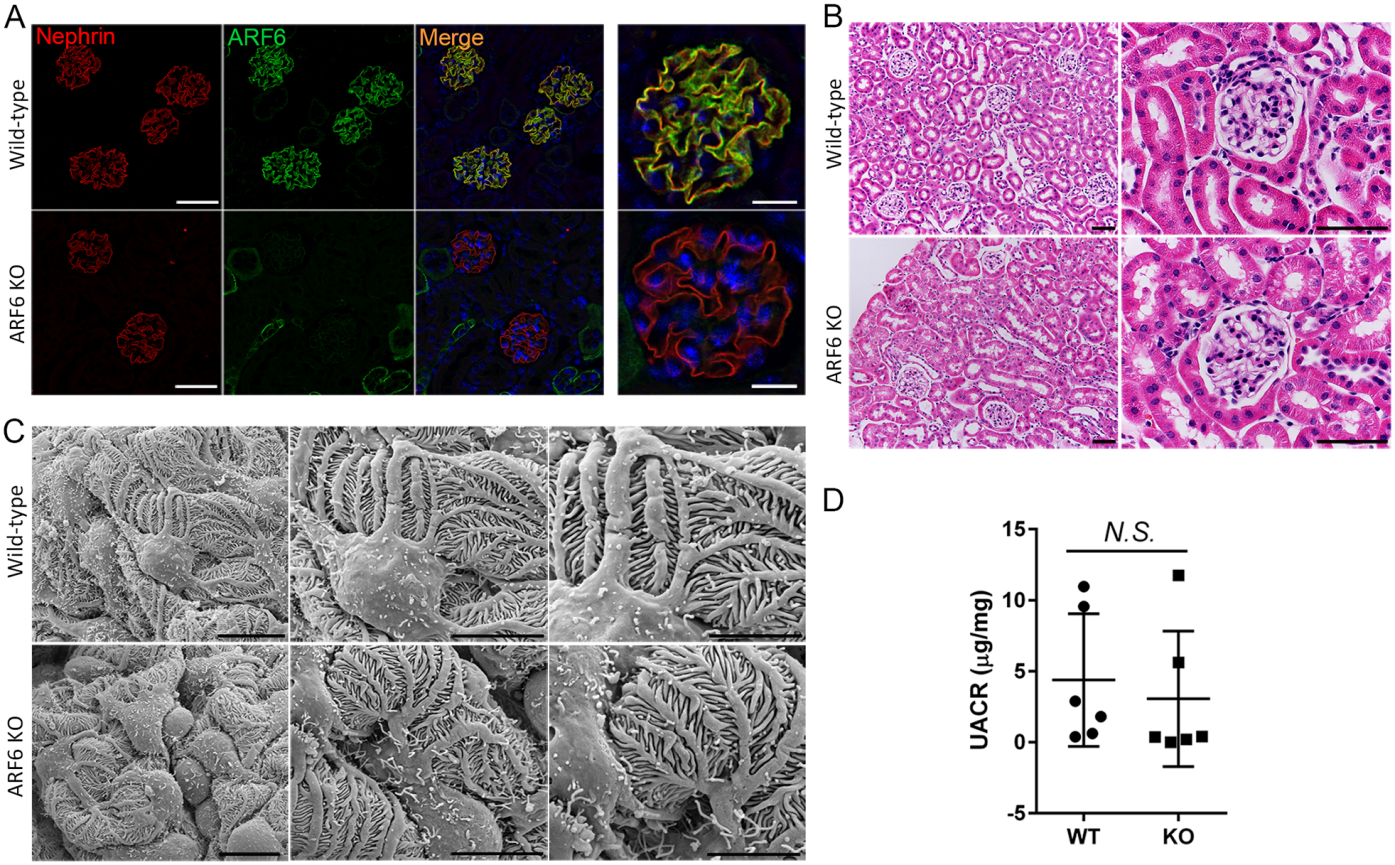
Podocyte-specific ARF6 null mice have no obvious renal abnormalities. The *Arf6* locus was targeted by homologous recombination with loxP sites flanking exon 1 and 2. Floxed *Arf6* mice were bred with *NPHS2*-Cre (podocin-Cre) mice to obtain podocyte-specific ARF6 knockout (*Arf6*^f/f^;*Nphs2*-Cre^Tg/+^) mice. Mice homozygous for floxed *Arf6* allele, but lacking *Nphs2*-Cre allele were used as control (*Arf6*^f/f^;*Nphs2*-Cre^+/+^). A. Indirect immunofluorescence microscopy of nephrin (red) and ARF6 (green) staining demonstrated podocyte-specific ARF6 deletion. Magnification (left) x40, scale bar 60 μm; (right) x63, scale bar 20 μm. B. Mouse kidney tissue sections were stained with Periodic acid-Schiff and examined by light microscopy. Magnification (left) x20, scale bar 60 μm. Magnification (right) x60, scale bar 60 μm. C. Glomerular ultrastructure was assessed by scanning electron microscope. Magnification (left) x3000, scale bar 10 μm; (middle) x6000, scale bar 10 μm; (right) x10000, scale bar 5 μm. D. Urine albumin to creatinine ratio was determined for 12-month-old podocyte-specific ARF6KO and control mice.

We examined the functional necessity of ARF6 in podocyte development and response to injury as our data suggested that ARF6 deletion should protect against injury-triggered nephrin tyrosine phosphorylation-mediated podocyte effacement. To analyze the *in vivo* function of ARF6 in podocytes, we selectively deleted the *Arf6* gene from podocytes in mice homozygous for the floxed *Arf6* allele and heterozygous for *Nphs2*-Cre (podocin-Cre) allele to generate podocyte-specific ARF6 knockout mice (ARF6_PodKO, *Arf6*^f/f^;*Nphs2*-Cre^Tg/+^). Mice homozygous for floxed *Arf6* allele, but lacking *Nphs2*-Cre allele were used as control (ARF6_WT, *Arf6*^f/f^;*Nphs2*-Cre^+/+^). See Methods for detailed description of podocyte-specific ARF6 knockout mice generation (S3 Fig) [50]. Indirect IMMUNOFLUORESCENCE microscopy staining with anti-nephrin and anti-ARF6 confirmed podocyte-specific ARF6 deletion (Fig 6A). ARF6 offspring on a mixed background were born at the expected Mendelian frequency, and behaved and aged normally when followed out to ≥ 1 year. Morphological exam by light and scanning electron microscopy (SEM) of glomeruli observed over 12 months revealed no alterations in ARF6_PodKO relative to control (Fig 6B and 6C). Development of spontaneous proteinuria was not detected with aging to 12 months (Fig 6D).

### Podocyte-specific ARF6 null mice were protected from protamine sulfate-induced foot process effacement

**Fig 7:**
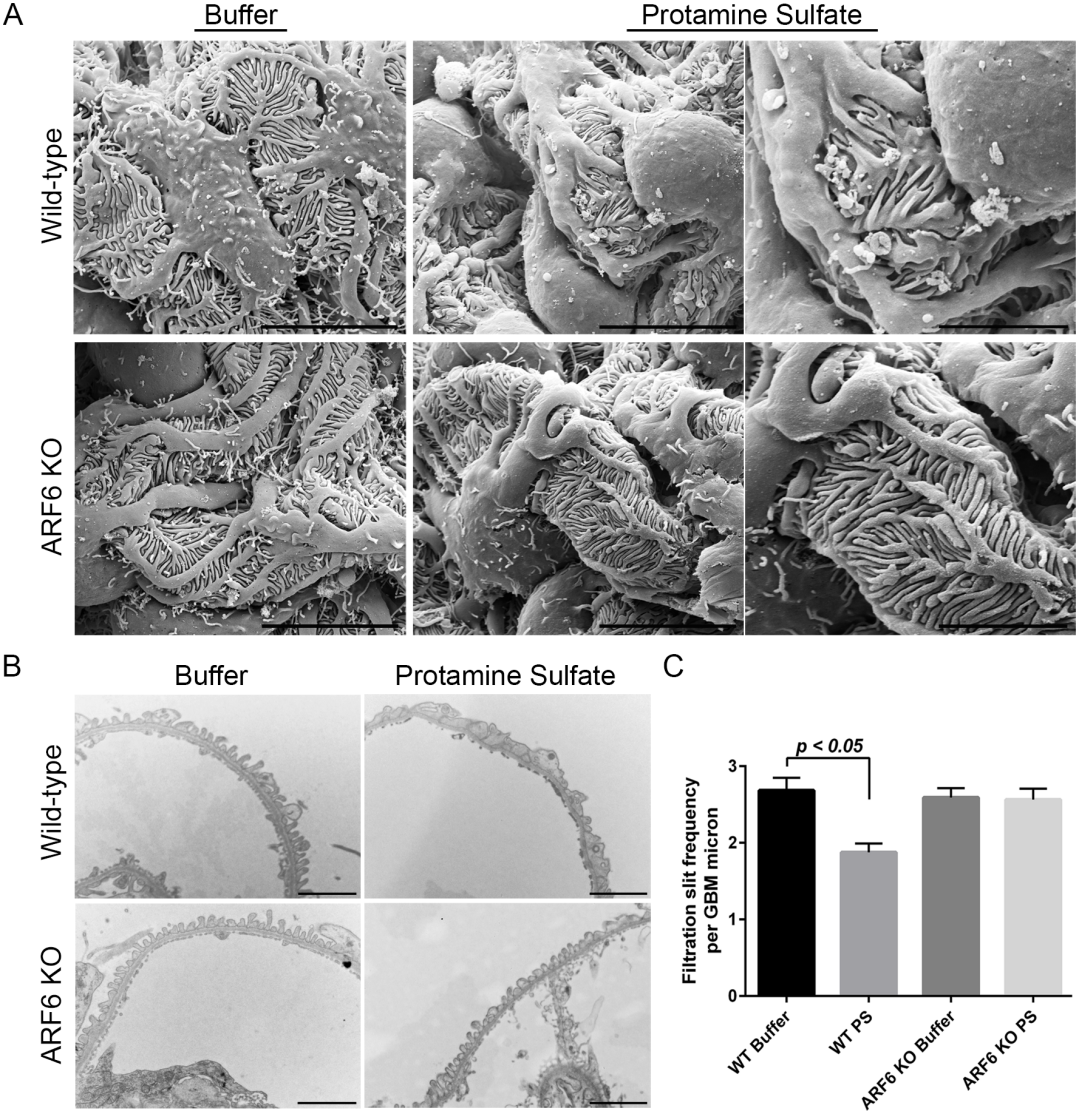
Podocyte-specific ARF6 null mice were protected from protamine sulfate-induced foot process effacement. A. Protamine sulfate (PS) perfusion was performed in WT (*Arf6*^f/f^; *Nphs2*-Cre^+/+^) and podocyte-specific ARF6 null (*Arf6*^f/f^;*Nphs2*-Cre^Tg/+^) mice. Hank’s balanced salt solution was used as control buffer perfusion. Podocyte morphology was evaluated by scanning electron microscope. Magnification (left) x6000, scale bar 10 μm; (middle) x6000, scale bar 10 μm; (right) x10000, scale bar 5 μm. B. Foot process alterations were evaluated by transmission electron microscope. Magnification x10000, scale bar 2 μm. C. The number of intercellular junctions per glomerular basement membrane (GBM) length was compared between all groups of mice (mean ± SEM). Results are representative of 4–6 mice per group.

Since ARF6 was necessary for nephrin activation-mediated podocyte spreading in culture, we speculated that the functional loss of ARF6 might become evident after challenging these mice to protamine sulfate (PS) perfusion. The PS model is an acute podocyte injury model that triggers nephrin tyrosine phosphorylation and results in rapid foot process effacement within minutes of renal artery perfusion [24, 26, 40, 46, 51–54]. PS-induced injury can be reversed by perfusion with heparan sulfate solution; consequentially this terminal model is frequently used to study podocyte actin dynamic-related signaling mechanisms. We perfused ARF6_PodKO and ARF6_WT mice with PS or buffer (control). Following PS perfusion, ARF6_WT mice showed extensive foot process spreading, while remarkably, ARF6_PodKO mice were protected from injury-induced effacement (Fig 7A and 7B). Foot process alterations were quantified by transmission EM. Buffer-perfused ARF6_WT and ARF6_PodKO mice had 2.7 ± 0.16 and 2.6 ± 0.12 slit diaphragm junctions per glomerular basement membrane (GBM) micron, respectively. PS-perfused ARF6_WT mice had 1.8 ± 0.12 slit diaphragm junctions per GBM micron compared to 2.6 ± 0.14 slit diaphragm junctions per GBM in PS-perfused ARF6_PodKO mice (Fig 7B and 7C). These results are consistent with the conclusion that ARF6 is necessary for injury-induced nephrin tyrosine phosphorylation signaling-mediated podocyte effacement.

### Nephrotoxic serum nephritis-induced injury resulted in aggravated proteinuria following podocyte-specific ARF6 deletion

**Fig 8:**
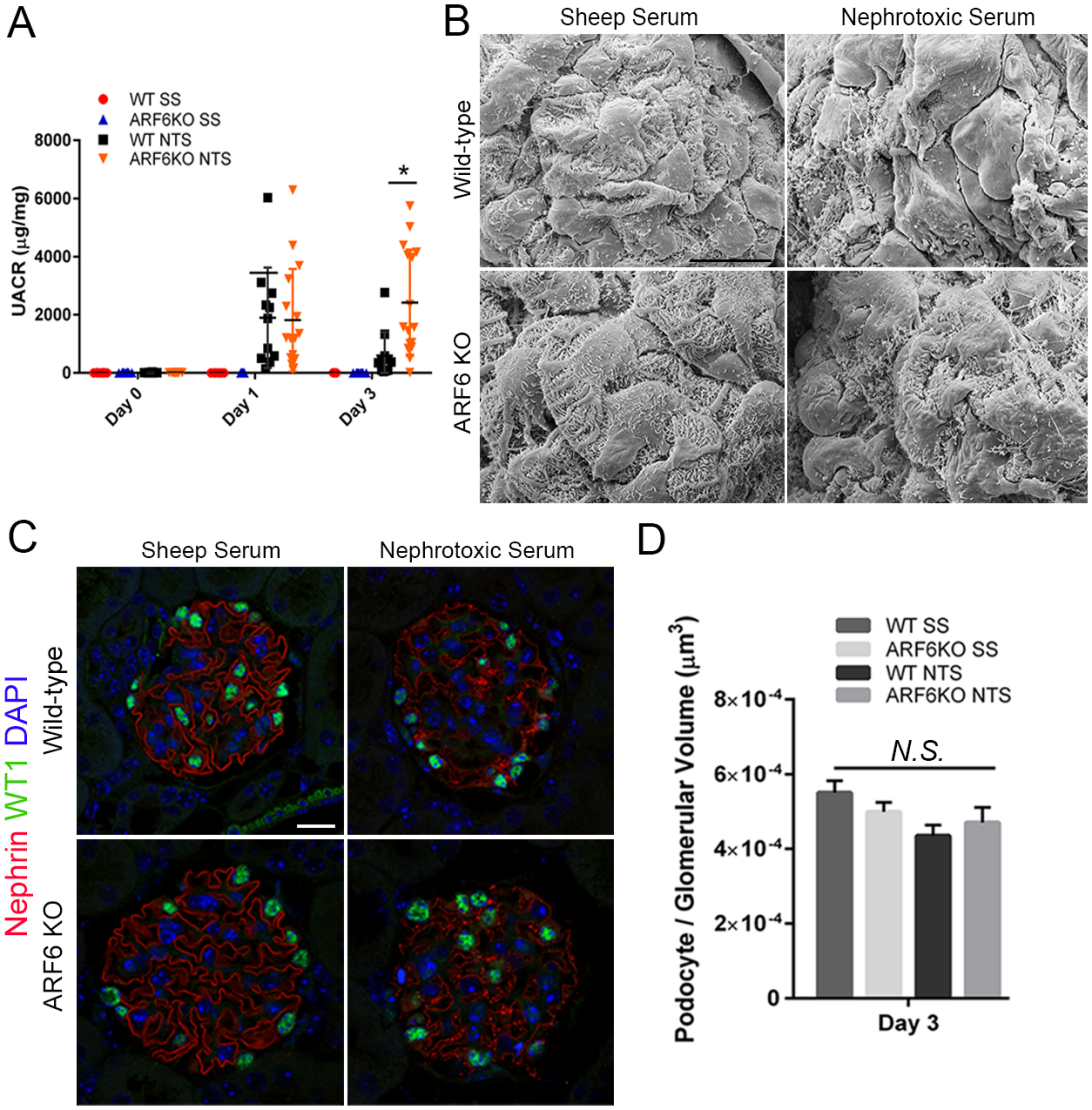
Podocyte-specific ARF6 null mice have aggravated proteinuria following nephrotoxic serum nephritis glomerular injury. A. Urine albumin to creatinine ratio (UACR) was determined for sheep serum or nephrotoxic serum (NTS) treated WT and podocyte-specific ARF6 null mice (mean ± SEM). Results are representative of 10–15 mice per group. B. Day 3 glomerular ultrastructure was assessed by scanning electron microscope. Magnification x3000. Scale bar 5 μm. C. Indirect immunofluorescence of mouse kidney tissue sections stained with anti-WT1 (green) and anti-nephrin (red). Magnification x63. Scale bar 20 μm. D. Podocyte number per glomerular volume (μm^3^) in all treatment groups.

To determine whether the observations made in the PS model could be extended to a different injury model, we used the well-described nephrotoxic serum nephritis (NTS) model of glomerular injury [25, 40, 55]. Introduction of heterologous sheep anti-rat glomerular lysate antiserum (i.e. NTS) resulted in dosage-dependent immune-complex mediated acute kidney injury [56]. Proteinuria was dramatically increased in both NTS-treated ARF6_WT and ARF6_PodKO mice. At 24 hours following treatment, both NTS-treated ARF6_WT and ARF6_PodKO mice had similar urine albumin to creatinine ratios (UACR). Consistent with previous reports of this model, proteinuria decreased by day 3 in ARF6_WT mice treated with NTS. In contrast, proteinuria further increased at day 3 in treated ARF6_PodKO mice (Fig 8A).

Ultrastructural exam of day 3 glomeruli by electron microscopy revealed severe podocyte effacement that was indistinguishable between NTS-treated ARF6_WT and ARF6_PodKO mice (Fig 8B). Investigation of podocyte density by indirect IMMUNOFLUORESCENCE was unrevealing (Fig 8C and 8D). While deletion of ARF6 in the PS model protected against podocyte effacement, the results from the NTS model suggests that deletion of ARF6 does not attenuate podocyte damage in all models of glomerular injury where distinct injury-activated signaling pathways may determine whether ARF6 activity is beneficial or deleterious [35, 57–59].

## Discussion

Earlier work established that podocyte injury activates nephrin signaling resulting in effacement in which PI3K and Rac1 are necessary [22, 24, 45, 46]. ARF6 has a key role in actin organization in coordination with Rac1 in other cell models, but its expression in podocytes was previously unknown. In this study, we report the first validation of ARF6 expression in human podocytes and the following observations: in podocyte cell culture, ARF6 activity is necessary for nephrin tyrosine phosphorylation-mediated podocyte ruffling and focal adhesion turnover. ARF6 appears to be necessary for nephrin-activation induced Rac1 activity, a small GTPase activity associated with ruffling and focal adhesion turnover. While deletion of ARF6 in podocytes did not result in observable developmental or functional kidney defects with aging in mice, glomerular injury-specific mechanisms might dictate whether ARF6 activity has beneficial or deleterious effects on maintaining glomerular filtration barrier integrity.

We created ARF6_PodKO mice to confirm our *in vitro* observations and found that ARF6 was dispensable for mouse podocyte development; this observation was in contrast to previously published literature where ARF6 was necessary for mouse hepatic cord and oligodendrocyte development [57, 60]. Multiple ARF, ARL (ARF-like), ARFRP (ARF-related proteins), and SAR (secretion-associated and Ras-related) proteins have been reported [61–63]; these proteins might also be expressed in podocytes and functionally compensate for loss of ARF6 during podocyte development. As noted above, there is a known relationship between ARF6 and Rac1 and like podocyte-specific ARF6 null mice, Rac1 null mice also had no observable developmental or kidney abnormalities, whereas *in vivo* podocyte-specific deletion of CDC42, another Rho family small GTPase, resulted in proteinuria, effacement, and glomerulosclerosis [46]. Further work is necessary to understand the potentially complex relationships and roles of the ARF, ARL, ARFRP, and SAR members in podocyte biology [33, 35, 64, 65].

Although podocyte signaling pathways necessary for morphogenesis were not disrupted in ARF6_PodKO mice, these pathways might be distinct from those activated during glomerular injury. We investigated ARF6 function in two acute injury models, protamine sulfate perfusion and NTS nephritis. Remarkably, following protamine sulfate perfusion ARF6_PodKO mice were protected against protamine sulfate-induced podocyte effacement [24, 26, 40, 54]. These observations were consistent with our *in vitro* experimental data that deletion of ARF6 in podocytes inhibits nephrin activation-induced cell ruffling and focal adhesion turnover, potentially by altering Rac1 activity.

Our observation that ARF6 deletion protects against PS-induced effacement is also consistent with work published by Blattner *et al*. that reported that podocyte-specific Rac1 null mice were protected from PS-induced podocyte effacement [46]. An ARF6-Rac1 functional relationship has been examined in other cell model systems where ARF6 directly and indirectly regulated Rac1 activity through lipid composition modulation [66–70], activation and recruitment of Rac1 GEFs to Rac1 in migrating epithelia and Madin-Darby canine kidney cells [37, 39, 49, 70–75], and recycling Rac1 raft domains to the plasma membrane [76, 77]. The functional specificity of ARF6 might be determined by the composition of associated small GTPase regulatory proteins (i.e. ARF-GEFs and -GAPs) [61, 64, 78]. For example, human gene mutations in Rac1 regulatory proteins, *ARHGAP24* and *ARGHDIA*, have been reported to alter Rac1 activity and are associated with focal segmental glomerulosclerosis [12, 29–31]; more so, recent evidence suggests that these disturbances are implicated in minimal change disease [12]. Identification of ARF6’s role in podocyte injury responses adds to this complexity. Additional studies are necessary to delineate the podocyte-specific ARF6-Rac1 biochemical and functional relationships, which might reveal therapeutic targetable elements.

Particular mouse glomerular injury models mimic elements of specific human glomerular diseases: the NTS model mimics elements of human immune complex-mediated glomerulonephritis [13, 55], while, arguably, the PS model might be more relevant to human minimal change disease [79]. For this reason, we examined a second mouse model of glomerular injury. Podocyte-specific deletion of ARF6 in the NTS model actually aggravated, rather than attenuated, the development of proteinuria following injury. While significant differences were not observed between NTS-treated ARF_WT and ARF6_PodKO mice by glomerular ultrastructure examination or by quantifying podocyte density, these findings nevertheless suggest that ARF6 has pleiotropic intracellular functions where deletion of ARF6 is not beneficial in all cases of podocyte injury. This is consistent with our previous observations that signaling pathways activated in human glomerular diseases can be distinct. For example, phosphorylated focal adhesion kinase and phosphorylated p130^Cas^, two signaling components necessary for altering nephrin-dependent cytoskeletal dynamics, were induced in patients with minimal change disease, but not in patients with focal segmental glomerulosclerosis [25]. Perhaps alteration of ARF6 activity is only relevant in a specific subset of glomerular disease patients.

In summary, we show that ARF6 expression is enriched in human podocytes and is necessary for nephrin tyrosine phosphorylation-induced podocyte cytoskeletal dynamics in cell culture and that it influences podocyte behavior in two distinct mouse podocyte injury models. Additional studies are necessary to delineate the podocyte ARF6-Rac1 relationship and disease-specific functional roles of ARF6. Understanding these mechanisms might yield therapeutic targets for human glomerular diseases such as minimal change disease or focal segmental glomerulosclerosis.

## Materials and methods

### Antibodies

Rabbit polyclonal antibodies against mouse nephrin and mouse phospho-nephrin^Tyr1191/Tyr1208^ were produced in our lab as previously described [14]. The following commercial primary antibodies and dilutions were used: rabbit anti-mouse phospho-nephrin^Tyr1176+1193^ (1:1000, ab80299, Abcam), anti-nephrin guinea pig polyclonal serum (1:500, GP-N2, Progen Biotechnik); rabbit polyclonal to ARF6 (1:200, ab77581, Abcam), rabbit polyclonal antibody against phospho-paxillin^Tyr118^ (1:200, #2541, Cell Signaling); rabbit polyclonal antibodies against vinculin (1:200, ab73412, Abcam); rabbit anti-lamellipodin (1:400, HPA016744, Sigma-Aldrich), rabbit monoclonal antibody against WT1 (1:100, Sc-19, Santa Cruz), and mouse anti-β-actin (1:500, A5441, Sigma-Aldrich).

### Cell culture and transductions

Conditionally immortalized human i.e. wild-type podocytes, a gift from Dr. Moin Saleem (University of Bristol, Bristol, United Kingdom) were cultured as described previously [21, 22, 71]. Plasmid constructs were generated in which the extracellular CD16 (clonal domain 16) and the CD7 transmembrane domain were fused with WT nephrin cytoplasmic domain (CD16/7-nephrin). Transient transduction of CD16/7-nephrin was performed using BacMam virus (final conc at 1% v/v, GlaxoSmithKline) for 24 hours.

Transient knockdown of endogenous ARF6 (ARF6KD) in human podocytes was performed using prepackaged lentivirus-based shRNA: lentiviral ARF6 shRNA and control shRNA not matching human genes (5 μl/12-well plate, 10^6^ TU, Mission shRNA Lentiviral Transduction Particles, Sigma). Stable human ARF6KD podocyte cell lines were created by addition of puromycin (2.5 μg/ml) to podocytes infected with ARF6 Mission shRNA Lentiviral Transduction Particles (TRCN0000048005, Sigma-Aldrich). After two weeks puromycin-resistant cells were collected and immunoblot assay was used to verify the KD of ARF6. pARF6-CFP (#11382), pARF6 (Q67L)-CFP (#11387), and pARF6 (T27N)-CFP (#11386) were obtained from Addgene (gifts from Joel Swanson, Cambridge, MA, USA).

To prepare an expression vector for Arf6 shRNA rescue experiments, a mutant human ARF6-CFP construct with 4 nucleotide silent (same-sense) mutations in the above ARF6 shRNA target region (**a**ct**t**ac**t**tggtt**g**acctctaa) (mtARF6-CFP) was created using QuikChange^®^ II mutagenesis kit (Agilent).

### CD16/7-nephrin activation model

The CD16/7-nephrin activation model allows for precise induction of nephrin tyrosine phosphorylation through addition of mouse anti-CD16 antibody and a fluorescent secondary anti-mouse IgG antibody to the culture media of live cells resulting in clustering CD16/7-nephrin and tyrosine phosphorylation—this model enables nephrin activation-mediated podocyte signaling and cell dynamics to be studied in detailed [21, 22, 37]. As previously described, CD16/7-nephrin WT podocytes were starved in serum free media (SFM) for 4 hours, then cooled on ice for 5 min. Monoclonal mouse anti-CD16 antibody in cold SFM (final conc 4 μg/ml, BD biosciences, BDB555404) was added. After 30 min of incubation on ice, cells were washed with phosphate buffer saline (PBS) and pre-warmed secondary antibody either Texas Red conjugated (final conc 3 μg/ml in SFM for immunofluorescence; T862, Life Technologies) or unconjugated goat anti-mouse IgG (final conc 1 μg/ml in SFM for biochemistry assay; 31160, Pierce) to induce clustering of CD16/7-nephrin proteins and nephrin tyrosine phosphorylation. Cells were then incubated for the designated time periods (0–20 min) at 37°C. No secondary antibody was added to the 0 min time-point. Cells were then lysed or fixed for biochemical or immunofluorescence assays.

### Immunofluorescence in cells

Podocytes were fixed in 4% paraformaldehyde (PFA)/PBS for 10 min, washed with PBS, and permeabilized with 0.1% Triton X-100 in PBS for 10 min. Non-specific staining was blocked with 5% bovine serum albumin (BSA)/PBS. The fixed cells were then incubated with primary antibodies overnight at 4°C followed by secondary antibody Alexa 488-conjugated goat anti-rabbit IgG (1:2000, Invitrogen) for 1 hr at room temperature. For the 0 min time-point, Texas Red-conjugated goat anti-mouse IgG (1:2000 in 5% BSA/PBS) was used. Images were taken on Leica STED (stimulated emission depletion) 3x Super-resolution microscope system equipped with a 63x oil immersion objective lens (Cancer Development Biology Microscopy Core, University of Pennsylvania).

#### Quantification of lamellipodia

WT and stable ARF6KD cells were stained with rabbit anti-lamellipodin antibody overnight at 4°C then incubated with Alexa 488 (green) conjugated goat anti-rabbit IgG for 1 hour at room temperature, and mounted on glass slides with ProLong Gold Antifade reagent. CD16/7-nephrin expressing podocytes were identified by Texas Red stain. Transduced podocytes with protrusions typical of lamellipodia architecture identified by anti-lamellipodin (green) staining were counted as positive for with lamellipodia. CD16/7-nephrin lamellipodia-positive cells over total CD16/7-nephrin cells was determined as percent of podocytes with lamellipodia. Human podocytes were co-transfected with equal amounts of CD16/7-nephrin and pARF6-CFP, pARF6 (Q67L)-CFP, or pARF6 (T27N)-CFP using Lipofectamine 2000 (Invitrogen). After 24 hours, the cells were stimulated using primary anti-CD16 Ab on ice followed by secondary Ab at 37°C for 20 min. CD16/7-nephrin expressing WT podocytes were identified by Texas Red stain and pARF6 by cyan fluorescence. The transfected cells revealed by mtARF6-CFP were taken using Zeiss laser scanning confocal microscopy. Quantification was performed using a blind experimental procedure by an uninformed observer on coded samples. Over 100 nephrin-positive cells were counted per condition [21, 22].

#### Quantification of focal adhesions

Cells were stained for p-paxillin or vinculin antibody and imaged by indirect immunofluorescence microscopy, as described above to identify FA. These images were analyzed using image thresholding techniques enabled by Image J 1.49p plugin “*Analyze Particles*” [72]. FA lengths were defined by Feret’s diameter i.e. the longest distance between any two points along the selection boundary [73, 74]. After determining the cell surface area, the number of FA ≥ 5 μm / 100 um^2^ cell surface area was quantified. Approximately 30 cells were analyzed per condition.

### Immunoblot

Cultured podocytes were lysed in modified RIPA buffer (50 mM HEPES pH 7.5, 150 mM NaCl, 1% NP-40, 0.1% SDS, 0.5% sodium deoxycholate, 1.5 mM MgCl_2_, 1 mM EGTA pH 8.0, 10% glycerol) containing protease and phosphatase inhibitor cocktails (Roche), and then centrifuged at 12,000 *rpm* for 20 min. Lysates (30 μg/sample) were ran on sodium dodecyl sulfate polyacrylamide gel electrophoresis (SDS-PAGE), transferred to nitrocellulose membranes (GE Healthcare Bioscience), blocked for 1 hour in 5% non-fat milk or BSA in Tris-buffered saline containing 0.05% Tween-20 (TBS-T), incubated with primary antibodies overnight at 4°C, washed, incubated with horseradish peroxidase (HRP)-conjugated secondary antibodies for 1 hour at room temperature, and then developed using ECL chemiluminescence reagent (Pierce).

### Immunoprecipitation

Podocyte lysates (500 μg/sample) and primary antibodies (2 μg) were rotated end-over-end overnight at 4°C. Samples were then incubated with 50 μl of Protein G Sepharose 4B (GE Healthcare Bioscience) for 2 hours at 4°C. The precipitates were recovered after brief centrifugation, washed with 0.1% NP40/PBS, and suspended in 25 μl of 2x Laemmli Buffer containing 2-Mercaptoethanol (1.25%) for SDS-PAGE. IB was performed as above. Normal non-immune rabbit IgG (Alpha Diagnostics, San Antonio) was used as negative control. Trueblot HRP-conjugated goat anti-rabbit or goat anti-mouse IgG (eBioscience) was used to decrease background from IgG heavy and light chains.

### G-protein pulldown assays

ARF6 activity was determined using ARF6-GTP pull-down kit following manufacturer’s instructions (Arf6 Activation Assay Biochem Kit, Cytoskeleton). Briefly, following CD16/7-nephrin activation assay, cell lysates (500 μg) were incubated with GGA3-PBD beads (15 μl) for 1 hour at 4°C then centrifuged. Beads were washed, re-spun, and mixed with 20 μl of 2x Laemmli sample buffer. IB described as above.

Rac1 activity was measured using a Rac1-GTP pull-down kit following manufacturer’s instructions (Rac1 Activation Assay Biochem Kit, Cytoskeleton). Cell lysates (500 μg) were incubated with PAK-PBD beads (10 μl) for 1 hour at 4°C then centrifuged. Beads were washed, spun, and 20 μl of 2x Laemmli sample buffer was added. Samples were analyzed by IB.

### Generation of podocyte-specific ARF6 null mice

The initial vector used to generate flox ARF6 mice was created by the trans-NIH Knock-Out Mouse Project (KOMP) and obtained from the KOMP Repository (www.komp.org). We modified this initial ARF6 deletion vector (Project ID CSD27672) to generate our ARF6 conditional targeting vector. Briefly, using a BAC clone (RP24-109D18) obtained from BACPAC Resources Center at Children’s Hospital Oakland Research Institute as template, we first mutated two endogenous KflI RE sites of ARF6 to KpnI RE sites and added the loxP sequence and KflI RE site to its 5’ end and FRT sequence and ClaI RE site to its 3’ end by PCR. This 4.3 kb KfiI/ClaI DNA fragment was digested and ligated to KflI/ClaI digested KOMP ARF6 deletion vector. The initial vector contained a part of ARF6 exon 2 at its 3’ homologous arm. To rectify this, we replaced the 5 kb RsrII/PacI fragment with a 1124 bp RsrII/SnaBI/PacI fragment by PCR using RP24-109D18 BAC clone as our template. The inclusion of a unique SnaBI RE site by PCR allowed the insertion of a new 3’ arm. We generated a new 2436 bp SnaBI/PacI fragment by PCR and replaced the 398 bp SnaBI/PacI to complete our ARF6 conditional targeting vector (22516 bp). An18,836 bp Nsi/AbsI targeting fragment from our conditional vector was electroporated into passage P 15 V6.5 embryonic stem cells, a 129/B6 F1 hybrid line. Correctly targeted clones were microinjected into pseudo-pregnant C57BL/6J females to obtain chimeras by standard procedures. The chimeric founders were bred to transgenic ACTB-Flpe mice to remove the neo cassette and to generate flox ARF6 offsprings. Heterozygous ARF6^f/+^ mice were back-crossed 2 generations to C57BL/6J mice carrying *Nphs2*-cre transgene; these N2 mice were intercrossed to obtain homozygous ARF6^f/f^ mice with or without *Nphs2*-cre allele for experiments conducted in this study (S2 Fig) [46]. Mice were housed in a specific pathogen free facility with 24-hour access to chow and water and a 12-hour day/night cycle. Breeding and genotyping was done according to standard procedures.

All animal studies were approved by the Institutional Animal Care and Use Committee at the University of Pennsylvania (Protocol #804511) and in accord with the National Institutes of Health *Guidelines for the Care and Use of Experimental Animals*.

### Glomerular stress models

#### Protamine sulfate perfusion

Perfusion of mice was carried out at 12 weeks of age as previously described [11, 20, 22, 42, 50]. Briefly, animals were anesthetized and perfused with PS (Sigma, P4380) in Hank’s balanced salt solution (HBSS) or HBSS alone (control buffer perfusion). Tissue was fixed with 4% PFA/PBS for immunohistochemistry analysis or glutaraldehyde-formalin for electron microscopy. For SEM, 10–15 glomeruli were analyzed per sample using a Philips XL20 scanning electron microscope (Electron Microscopy Research Laboratory Core, University of Pennsylvania). For TEM, samples were examined with a JEOL 1010 electron microscope (Electron Microscopy Research Laboratory Core, University of Pennsylvania). Five to 10 glomeruli per animal (n = 4-6/group) were evaluated and slit diaphragm frequency was assessed quantitatively by counting the number of junctions per micron of GBM using Image J software.

#### Nephrotoxic serum nephritis model

12-week-old mice were injected retro-orbitally with sheep anti-rat glomerular lysate antiserum, otherwise known as nephrotoxic serum (NTS) or sheep serum (control) at a concentration of 1.5 mg per mouse (approximately 25 g body weight, n = 10–15 mice/group). The characteristics of whole NTS were described previously [21, 36, 51]. Collection of spot urine samples was performed between 7 to 10 am at defined time-points as indicated. Urine albumin to creatinine ratios were measured using a mouse Albuwell and creatinine kit (Exocell, Philadelphia, PA) and an enzyme-linked immunosorbent assay plate reader (Beckman-Coulter DTX 880 reader, Indianapolis, IN).

### Immunofluorescence for kidney tissue sections

Paraffin-embedded sections (4 μm) were cut, deparaffinized, and rehydrated through graded alcohol. Antigen retrieval was achieved by heating sections at 98°C for 45 min in Tris-EDTA buffer (pH 9.0). Tissue was permeabilized with 1% SDS/PBS for 10 min, blocked for 1 hour in 10% goat serum, and incubated with primary antibodies overnight at 4°C. After five PBS-T washes, Alexa Fluor conjugated secondary antibodies were added to the sections for 1 hour at room temperature. Slides were then mounted using ProLong Anti-fade. Images were taken by a Leica STED 3x Super-resolution confocal microscopy system (Cancer Development Biology Microscopy Core, University of Pennsylvania).

For biopsy samples, normal human kidney tissue sections were obtained as de-identified human autopsy material. Tissue sections from these biopsies were subjected to double immunofluorescent staining for ARF6 and nephrin, and immunohistochemistry.

#### Podocyte counting

Sections of whole mice kidney tissue were prepared as described above and stained with anti-WT1, anti-nephrin, and DAPI mounting medium. Indirect IMMUNOFLUORESCENCE microscopy pictures were obtained. Podocyte number, glomerular area, and podocyte nuclei diameter were measured using ImageJ. Estimation of podocyte number and density using a single histologic section was determined as described by Venkatareddy M. et al [75]. Thirty to 50 glomeruli per mouse was assessed (n = 4 mice per group).

### Statistical analysis

Data are presented as mean ± SEM. Statistical evaluation was performed by using One-Way ANOVA with Dunnett’s multiple comparison posttests for comparing multiples or Two-Way ANOVA with Tukey’s multiple comparison posttests for comparing multiple groups (GraphPad Prism 4.0). A *p* < *0*.*05* was considered significant.

## Supporting information

S1 Fig ARF6 activity in podocytes is necessary for nephrin-activation induced lamellipodia. Podocyte ruffling assessed by anti-lamellipodin immunofluorescence stain (blue) following activation of CD16/7-nephrin (red) expressing WT and stable ARF6KD human podocytes. White arrow demonstrates cellular ruffling. B. Podocyte ruffling activity assessed by anti-lamellipodin (blue) 20 minutes following activation of CD16NCD (red) in stable ARF6KD human podocytes rescued by CFP-tagged mutant ARF6 (mtARF6) that resists to *ARF6* shRNA (green). C. Ruffling activity assessed by anti-lamellipodin (blue) 20 minutes following activation of CD16/7 expressing constitutively-active ARF6, ARF6(Q67L), or dominant-negative, ARF6(T27N) podocytes. Magnification x63. Scale bar 32 μm.
(TIF)

S2 Fig Expression of mtARF6 is verified in HEK293 cells co-transfected with ARF6-shRNA and mtARF6. A. Sequencing results showing that ARF6-shRNA target sites are mutated (arrow) in mtARF6. B. Hela cells were transfected with control shRNA, ARF6-shRNA alone or ARF6-shRNA plus CFP-tagged mtARF6. 48 hours following transfection, total cellular lysates were obtained and immunoblot was performed for ARF6 expression. *Arrow*: *endogenous ARF6*, *Asterisk*: *CFP-tagged mtARF6*.
(TIF)

S3 Fig Podocyte-specific ARF6KO mouse generation. ARF6-flox mice with LoxP sites flanking exon 1 and 2 were crossed with Cre mice in which Cre recombinase was driven by the podocyte-specific podocin promoter (*Nphs2*-Cre) generating *Arf6*^f/f^;*Npsh2*-Cre^Tg/+^ (podocyte specific-ARF6 null mice) and *Arf6*^f/f^;*Nphs2*-Cre^+/+^ (control mice, see detailed description in Methods). A. Schematic representations of the wild-type *Arf*6 allele, the targeting vector (neo), the floxed *Arf6* (f) allele and null *Arf6* allele. Exons are represented by filled box and the *Arf6* coding sequence within exon 2 is indicated by a red line. Arf6 neo mice were crossed with Flpase mouse to generate mice with deletion of genomic region within the two flp sites, indicated by green triangles. Floxed *Arf6* mice were crossed with *Nphs2*-Cre mice to generate podocyte specific-ARF6 null mice. LoxP sites are represented by filled red triangles. Locations of primers used for genotyping the ARF mice by PCR are shown. B. PCR analysis of mouse genomic DNA to screen for different *Arf6* alleles. β-globin amplicons served as an internal control.
(TIF)
