# Supplementary figures and images for: ARF6 mediates nephrin tyrosine phosphorylation-induced podocyte cellular dynamics
Source: PLoS One. 2017 Sep 7;12(9):e0184575. doi: 10.1371/journal.pone.0184575 (PMC5589247; doi:10.1371/journal.pone.0184575)

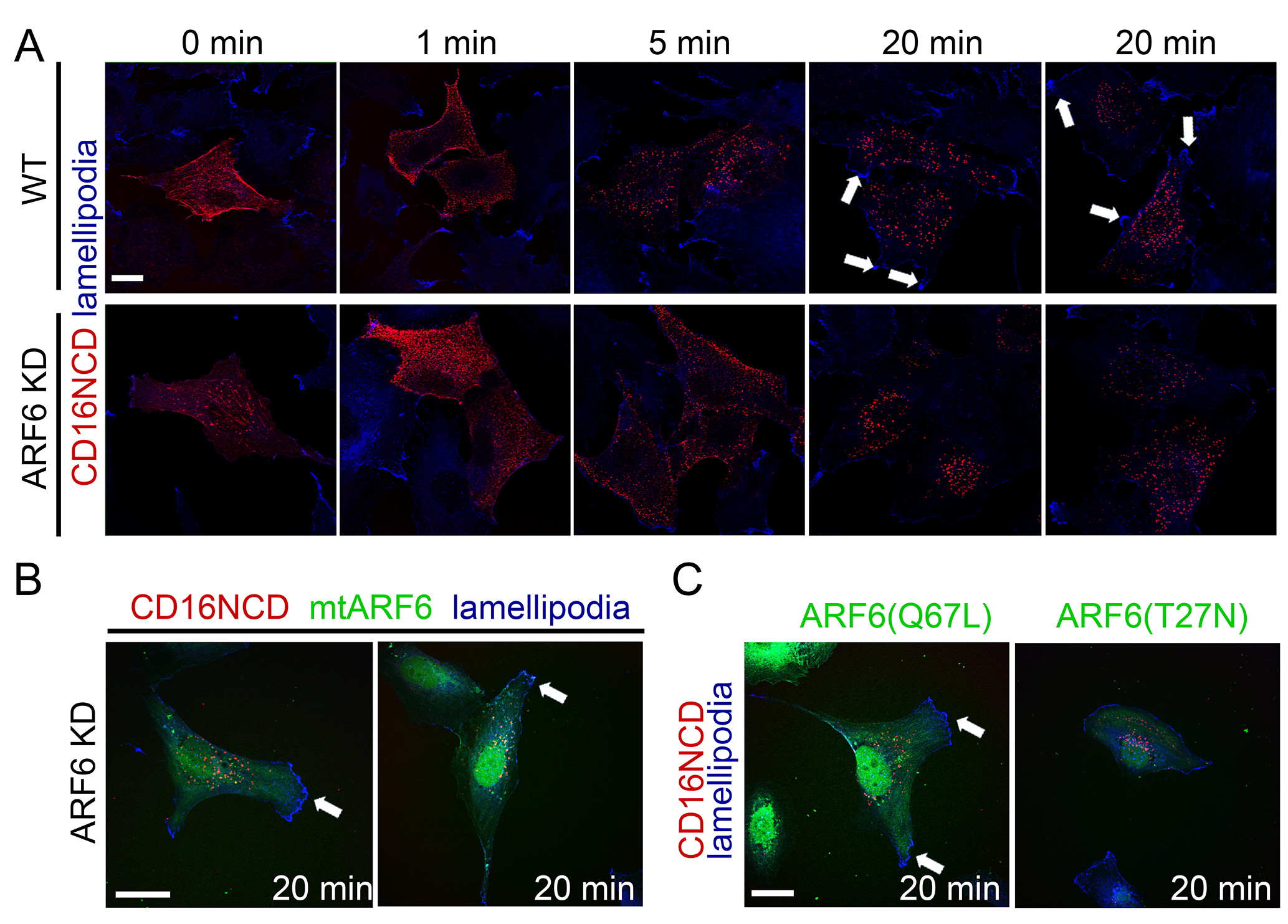

Supplement: S1 Fig — Podocyte ruffling assessed by anti-lamellipodin immunofluorescence stain (blue) following activation of CD16/7-nephrin (red) expressing WT and stable ARF6KD human podocytes. White arrow demonstrates cellular ruffling. B. Podocyte ruffling activity assessed by anti-lamellipodin (blue) 20 minutes following activation of CD16NCD (red) in stable ARF6KD human podocytes rescued by CFP-tagged mutant ARF6 (mtARF6) that resists to ARF6 shRNA (green). C. Ruffling activity assessed by anti-lamellipodin (blue) 20 minutes following activation of CD16/7 expressing constitutively-active ARF6, ARF6(Q67L), or dominant-negative, ARF6(T27N) podocytes. Magnification x63. Scale bar 32 μm. (TIF) [file pone.0184575.s001.tif]

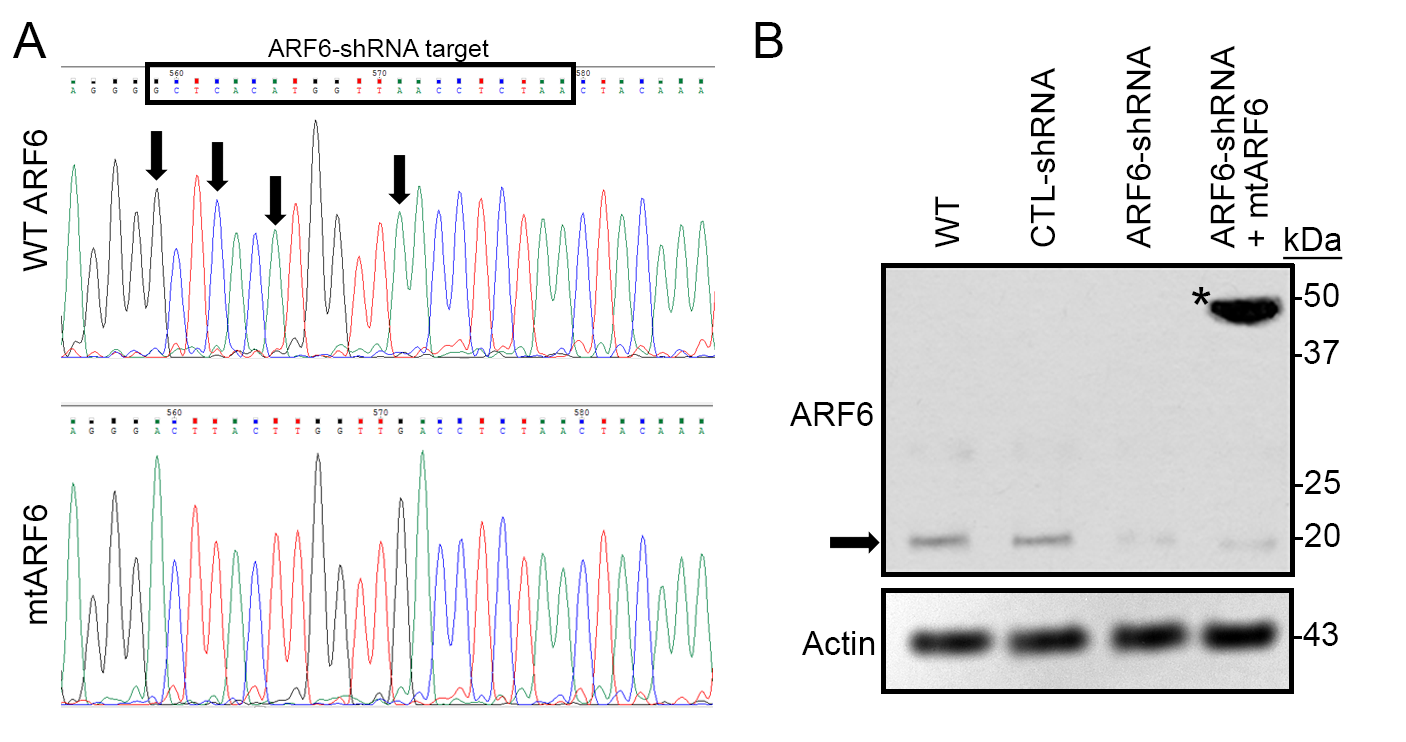

Supplement: S2 Fig — A. Sequencing results showing that ARF6-shRNA target sites are mutated (arrow) in mtARF6. B. Hela cells were transfected with control shRNA, ARF6-shRNA alone or ARF6-shRNA plus CFP-tagged mtARF6. 48 hours following transfection, total cellular lysates were obtained and immunoblot was performed for ARF6 expression. Arrow: endogenous ARF6, Asterisk: CFP-tagged mtARF6. (TIF) [file pone.0184575.s002.tif]

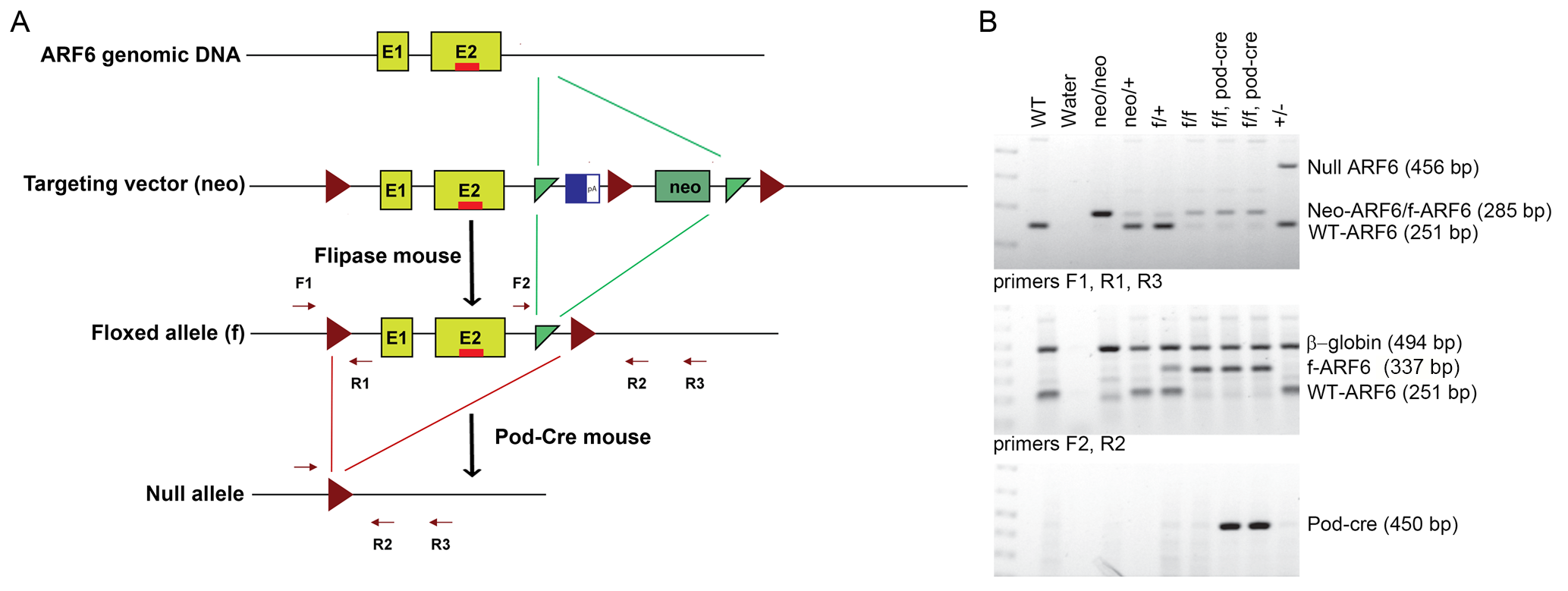

Supplement: S3 Fig — ARF6-flox mice with LoxP sites flanking exon 1 and 2 were crossed with Cre mice in which Cre recombinase was driven by the podocyte-specific podocin promoter (Nphs2-Cre) generating Arf6f/f;Npsh2-CreTg/+ (podocyte specific-ARF6 null mice) and Arf6f/f;Nphs2-Cre+/+ (control mice, see detailed description in Methods). A. Schematic representations of the wild-type Arf6 allele, the targeting vector (neo), the floxed Arf6 (f) allele and null Arf6 allele. Exons are represented by filled box and the Arf6 coding sequence within exon 2 is indicated by a red line. Arf6 neo mice were crossed with Flpase mouse to generate mice with deletion of genomic region within the two flp sites, indicated by green triangles. Floxed Arf6 mice were crossed with Nphs2-Cre mice to generate podocyte specific-ARF6 null mice. LoxP sites are represented by filled red triangles. Locations of primers used for genotyping the ARF mice by PCR are shown. B. PCR analysis of mouse genomic DNA to screen for different Arf6 alleles. β-globin amplicons served as an internal control. (TIF) [file pone.0184575.s003.tif]
